# Nitrite-Mediated Hypoxic Vasodilation Predicted from Mathematical Modeling and Quantified from *in Vivo* Studies in Rat Mesentery

**DOI:** 10.3389/fphys.2017.01053

**Published:** 2017-12-13

**Authors:** Donald G. Buerk, Yien Liu, Kelly A. Zaccheo, Kenneth A. Barbee, Dov Jaron

**Affiliations:** School of Biomedical Engineering, Science and Health Systems, Drexel University, Philadelphia, PA, United States

**Keywords:** aldehyde oxidoreductase, allopurinol, hypoxic vasodilation, nitrite reductases, nitric oxide, raloxifene, xanthine oxidoreductase

## Abstract

Nitric oxide (NO) generated from nitrite through nitrite reductase activity in red blood cells has been proposed to play a major role in hypoxic vasodilation. However, we have previously predicted from mathematical modeling that much more NO can be derived from tissue nitrite reductase activity than from red blood cell nitrite reductase activity. Evidence in the literature suggests that tissue nitrite reductase activity is associated with xanthine oxidoreductase (XOR) and/or aldehyde oxidoreductase (AOR). We investigated the role of XOR and AOR in nitrite-mediated vasodilation from computer simulations and from *in vivo* exteriorized rat mesentery experiments. Vasodilation responses to nitrite in the superfusion medium bathing the mesentery equilibrated with 5% O_2_ (normoxia) or zero O_2_ (hypoxia) at either normal or acidic pH were quantified. Experiments were also conducted following intraperitoneal (IP) injection of nitrite before and after inhibiting XOR with allopurinol or inhibiting AOR with raloxifene. Computer simulations for NO and O_2_ transport using reaction parameters reported in the literature were also conducted to predict nitrite-dependent NO production from XOR and AOR activity as a function of nitrite concentration, PO_2_ and pH. Experimentally, the largest arteriolar responses were found with nitrite >10 mM in the superfusate, but no statistically significant differences were found with hypoxic and acidic conditions in the superfusate. Nitrite-mediated vasodilation with IP nitrite injections was reduced or abolished after inhibiting XOR with allopurinol (*p* < 0.001). Responses to IP nitrite before and after inhibiting AOR with raloxifene were not as consistent. Our mathematical model predicts that under certain conditions, XOR and AOR nitrite reductase activity in tissue can significantly elevate smooth muscle cell NO and can serve as a compensatory pathway when endothelial NO production is limited by hypoxic conditions. Our theoretical and experimental results provide further evidence for a role of tissue nitrite reductases to contribute additional NO to compensate for reduced NO production by endothelial nitric oxide synthase during hypoxia. Our mathematical model demonstrates that under extreme hypoxic conditions with acidic pH, endogenous nitrite levels alone can be sufficient for a functionally significant increase in NO bioavailability. However, these conditions are difficult to achieve experimentally.

## Introduction

The primary source of the nitrite anion (${\text{NO}}_{2}^{-}$) in mammalian systems is from the oxidation of nitric oxide (NO) produced by the L-arginine/NO enzymatic pathway in vascular endothelium by the O_2_-dependent endothelial isoform of NO synthase (eNOS). Although physiological effects of nitrite on the cardiovascular system have been known since 1880 (Reichert and Mitchell, 1880), the consensus view had been that nitrite is an inert byproduct of NO production. This viewpoint has changed radically in the past few decades with the emergence of abundant evidence that nitrite serves as a reversible storage reservoir for NO, which can restore NO bioavailability under certain physiological conditions. However, the mechanisms for recovering NO from nitrite are incompletely understood since the biochemical formation of NO metabolic byproducts and regulation of NO bioavailability is complex (Kim-Shapiro and Gladwin, 2014; Blood, 2017; Helms et al., 2017). Furthermore, the accurate measurement of nitrite and related nitrogen species in blood and tissue is technically difficult (MacArthur et al., 2007).

Infusion of sodium nitrite (NaNO_2_) into the bloodstream has been shown to cause vasodilation in humans, presumably due to conversion of nitrite to NO (Cosby et al., 2003; Dejam et al., 2007; Pluta et al., 2011). Evidence that inorganic nitrite anion therapy may have therapeutic effects for numerous pathological conditions, especially for treating cardiovascular disease, has been reviewed (Kevil et al., 2011; Omar et al., 2016; Blood, 2017), along with substantial experimental evidence for a protective effect from ischemia-reperfusion injury (e.g., see Table 1 in Blood, 2017). Pluta et al. (2011) report that 48 h of continuous IV infusion of NaNO_2_ is well tolerated in humans, with a maximal tolerable dose of 267 μg/kg/hr. Three of the 12 subjects in this clinical study showed some toxicity at doses of 445.7 μg/kg/hr with a significant decrease in mean arterial blood pressure by more than 15 mmHg in two subjects, and in one subject the methemoglobin level exceeded 5%. Earlier studies using much higher doses of nitrite reported incidences of severe hypotension and lethal methemoglobinemia (Weiss et al., 1937; Wilkins et al., 1937), which curtailed further interest in therapeutic applications for decades. Despite these observed negative effects, nitrite is an approved therapeutic antidote for cyanide and hydrogen sulfide poisoning (Lloyd, 1957; Smith and Gosselin, 1979). More recently, interest in using nitrite for therapeutic purposes has been resurrected. A search of clinicaltrials.gov using nitrite as a keyword presently lists 48 clinical trials that include nitrite as the study drug. Many more dietary studies evaluating the effect of oral nitrate supplements are also listed.

NO generated from nitrite through the deoxyhemoglobin nitrite reductase pathway in red blood cells (RBCs) is proposed to play a major role in hypoxic vasodilation (Gladwin, 2008; Gladwin et al., 2009). However, our previous mathematical model (Buerk et al., 2011a) for coupled NO and O_2_ transport around an arteriole predicted that only negligible amounts of NO could reach smooth muscle cells (SMC) in the vascular wall due to very strong scavenging of NO by hemoglobin (Hb) in RBCs. Azizi et al. (2005) used the analogy that the RBC is a “black hole” for NO—it can get in but can't get out. Our previous mathematical model predicted that substantially more NO could be derived from nitrite reductase activity in tissue compared with the deoxyhemoglobin nitrite reductase pathway (Buerk et al., 2011a). Our model prediction for the minor contribution of NO from the deoxyhemoglobin nitrite reductase pathway is consistent with a mathematical model by another group (Chen et al., 2008), which predicted that only picomolar levels of NO could be delivered to vascular SMC. Buerk et al. (2011b) has reviewed other mathematical modeling predictions and relevant experimental data in the literature with respect to several signaling pathways in the microcirculation that involve NO. In general, we found that mathematical predictions for NO values are often lower than reported from experimental measurements, and that very few models developed by other investigators include both the O_2_-dependance of NO production from eNOS and the inhibitory effect of NO on O_2_ consumption in tissue (coupled NO and O_2_ transport), which we always include in our models.

More recently, we developed an alternative deoxyhemoglobin nitrite reductase model to investigate whether dinitrogen trioxide (N_2_O_3_) can act as a stable intermediate to preserve NO (Liu Y. et al., 2016). The model is based on the assumption that N_2_O_3_ does not react in the bloodstream (Basu et al., 2007; Hopmann et al., 2011) and will only release NO after it homolyzes in tissue (Butler and Ridd, 2004). Our alterative model predicts that NO is rapidly released from RBC-generated N_2_O_3_ after it leaves the bloodstream, primarily in the endothelium, with a resulting increase in SMC NO in the vascular wall (Liu Y. et al., 2016). Furthermore, this reaction is enhanced at low blood PO_2_ and increases with acidic pH.

We did not include generation of NO by tissue nitrite reductase activity in our recent model (Liu Y. et al., 2016), since we were examining a theoretical mechanism that could spare NO generated in RBCs from strong scavenging by Hb. However, tissue nitrite reductase activity is hypothesized to be a significant source of NO, especially during hypoxia. Both *in vitro* and *in vivo* studies demonstrate that NO generation from nitrite in tissue is associated with the molybdoenzymes xanthine oxidoreductase (XOR) and aldehyde oxidoreductase (AOR) (Li et al., 2008; Webb et al., 2008; Golwala et al., 2009). For the present report, we conducted experiments to test the hypothesis that tissue nitrite reductases increase NO bioavailability and modulate vascular tone of arterioles (20–80 μm diameter range) in the rat mesentery microvasculature under varying PO_2_ and pH conditions. We also modified our previous mathematical models (Buerk et al., 2011a; Liu Y. et al., 2016) using available reaction kinetic parameters in the literature for the tissue nitrite reductases XOR and AOR (Maia and Moura, 2011; Maia et al., 2015) to predict NO changes in arteriolar SMC as a function of nitrite concentration, PO_2_ and pH.

## Methods

### Animals and animal care

All animals received humane care according to the criteria outlined in the Guide for the Care and Use of Laboratory Animals prepared by the National Academy of Sciences and published by the National Institutes of Health. All animal protocols were approved by the Institutional Animal Care and Use Committee at Drexel University. Every effort was made to minimize animal pain and suffering. Male Sprague-Dawley rats (250–300 g, aged 8 weeks) were kept one or two per cage in a temperature-controlled room at 28°C (thermoneutrality for rats) under a 12-h light/12-h dark cycle. All male subjects were used to avoid confounding effects of estrogen on eNOS.

### *In vivo* microcirculation studies

Exteriorized rat mesentery experiments were conducted under isoflurane anesthesia to measure perivascular NO with recessed microelectrodes, arteriolar diameter (D) from video imaging (Neild, 1989) (DiamTrak software purchased from Dr. T.O. Neild, Flinders Univ., Adelaide, Australia), tissue perfusion (relative volumetric RBC flow in capillaries; Bonner et al., 1981) by laser Doppler (LDF, Transonic model BLF22, Ithaca, NY), and small artery (~270 micron diameter) blood flow with an ultrasonic probe (Transonic model 420, Ithaca, NY). All physiological signals were sampled at 10 Hz with 12-bit accuracy using a computer-controlled data acquisition system. The DiamTrak output was filtered to remove occasional out of range artifacts using Excel, and smoothed with a running average filter. Arteriolar vasodilation was quantified in response to NaNO_2_ in the superfusion medium (Krebs-ringer bicarbonate buffer) bathing the mesentery equilibrated with either 5% or 10% O_2_ and 5% CO_2_ (normoxic solution) or zero O_2_ (95% N_2_) and 5% CO_2_ (hypoxic solution) at normal (pH = 7.4) or acidic pH (range 6.5–6.7) and maintained at 37°C. Typically, paired measurements were made for each arteriole, alternating NaNO_2_ exposures between normoxic or hypoxic solutions. The concentration of NaNO_2_ in the superfusate was varied up to 25 mM, exposing the preparation to NaNO_2_ for only short periods of time (typically 3 min duration).

In addition, some superfusion experiments were conducted to quantify arteriolar responses before and after inhibiting XOR with the pyrazolopyrimidine-based inhibitor allopurinol (3.4–6 mg/kg IP). Allopurinol dissolved in normal saline was delivered by a single intraperitoneal (IP) injection through a tube inserted into the abdominal cavity. In addition to NaNO_2_ exposures in the superfusion solution, *in vivo* experiments were also conducted with measurements taken after an acute IP injection of 3–6 mg/kg mg of NaNO_2_ while the mesentery was superfused with hypoxic solution at pH = 7.4. After recording control measurements for 3–4 arterioles, XOR oxidase was inhibited with allopurinol and measurements were repeated for the same arterioles. We also conducted studies using either superfusion or an acute IP injection of NaNO_2_ before and after inhibiting AOR with the estrogen receptor antagonist raloxifene (2.9–10 mg/kg IP).

### Mathematical model

NO and O_2_ transport were simulated in a microcirculatory arteriole and surrounding tissue model and solved for steady state conditions using finite element method numerical methods (COMSOL v5.3, Burlington, MA). Coupled non-linear partial differential equations for mass transport were written in cylindrical coordinates including the sum of reactions (R_i_) for all chemical species (C_i_ = O_2_, NO, ${\text{NO}}_{2}^{-}$)

(1) $$\begin{array}{r} \nabla \cdot \left(D_{i}\nabla C_{i}\right)-\nu \nabla C_{i}\pm \sum R_{i}=0 \end{array}$$

as detailed in our previous modeling efforts (Buerk et al., 2003, 2011a; Lamkin-Kennard et al., 2004a,b; Chen et al., 2006; Chen X. et al., 2007; Liu et al., 2017), where D_i_ is the diffusion coefficient for each species, and *v* is the fluid velocity profile in the lumen (assumed to be parabolic).

The model has five concentric cylindrical layers: (i) RBC core, radius = 13 μm, (ii) RBC-free plasma layer, 13 < r < 14 μm, width = 1 μm, (iii) endothelium, 14 < r < 15 μm, width = 1 μm, (iv) vascular wall smooth muscle cell (SMC) layer, 15 < r < 25 μm, width = 10 μm, and (v) perivascular tissue, 25 < r < 130 μm, width = 105 μm. Each layer was assumed to have homogenous properties with uniformly distributed reactions. Both convective and diffusive mass transports are included in the vessel lumen, with only diffusive transport in tissue. NO is produced in the endothelium by eNOS, and generated from nitrite in tissue by either XOR or AOR, or in blood from conversion of nitrite to N_2_O_3_ (Basu et al., 2007) by Hb in RBCs, with subsequent homolysis to release NO. The model includes O_2_-dependent NO production by eNOS, and inhibition of O_2_ consumption by NO, using parameters as described for one of our previous models (Chen et al., 2006). The present model now includes reactions for nitrite in blood or tissue, which are compared to a baseline simulation without XOR or AOR.

We modeled the reaction of nitrite with Hb in the bloodstream as

(2) $$\begin{array}{r} \frac{d\left[NO\right]}{dt}=\;k_{N}\left[Hb\right]\left[NO_{2}^{-}\right] \end{array}$$

where the bimolecular rate constant *k*_*N*_ was characterized as a function of blood PO_2_ using a modified Monod-Wyman-Changeux (MWC) model of allostery for the oxyhemoglobin equilibrium curve, as described by Rong et al. (2013a,b). We further modified this model (Liu Y. et al., 2016), adding the production of N_2_O_3_ from NO and nitrite-methemoglobin, catalyzed by the nitrous anhydrase activity of deoxyHb. The O_2_-dependent function for k_N_ in Equation (2) and the complete model parameters used in our simulation are summarized in Liu Y. et al. (2016).

In tissue, the reaction rate for nitrite reduction by XOR was characterized using a Michaelis-Menten equation with competitive inhibition by O_2_:

(3) $$\begin{array}{r} \nu_{Nitrite\;Reduction}=\frac{k_{cat}\left[NO_{2}^{-}\right]\left[XOR\right]}{K_{m}NO_{2}^{-}\left(1+\frac{\left[O_{2}\right]}{K_{m}O_{2}}\right)+\left[NO_{2}^{-}\right]} \end{array}$$

where the reaction parameters *k*_cat_, *K*_m_${\text{NO}}_{2}^{-}$, and *K*_m_O_2_ vary depending on tissue pH values (Li et al., 2008; Maia and Moura, 2011; Maia et al., 2015; see Table 1). XOR was assumed to be uniformly distributed in the endothelium, SMC layer, and perivascular tissue (Ray and Shah, 2005). Blood PO_2_ was varied between normoxic conditions (90 Torr) down to hypoxic levels (10 Torr). Simulations were performed with tissue XOR concentrations ranging between 0.03 and 0.3 μM as found in heart and liver, respectively, where tissue nitrite reduction has been shown to produce a functionally significant elevation in NO (Kim-Shapiro and Gladwin, 2014). The nitrite anion was assumed to be uniformly distributed in all perivascular tissue regions. Simulations were performed with nitrite varying between physiological (<2 μM; Li et al., 2008; van Faassen et al., 2010) to elevated (300 μM) concentrations. Simulations were solved at steady state with a relative tolerance for convergence of 0.001 and an absolute tolerance of 0.0001. The initial mesh for the computational domain consisted of 19,976 domain elements and 1,678 boundary elements. Meshing was calibrated such that further refinement did not change predicted NO concentration more than 0.01 nM.

**Table 1:** Physical parameters and rate constants used in the simulation.

**Parameter**	**Value(s)**	**References**
Tissue nitrite	0–300 μM	
Nitrite diffusion coefficient XOR concentration	410 μm^2^/s	Pinotti et al., 2002; Li et al., 2008; Kim-Shapiro and Gladwin, 2014
Heart	0.03 μM	
Liver	0.3 μM	
Reaction parameters at pH = 7.4		Maia and Moura, 2011; Maia et al., 2015
k_cat_	0.545 s^−1^	
K_m_${\text{NO}}_{2}^{-}$	1918 μM	
K_m_O_2_	24.3 μM	
at pH = 6.3		
k_cat_	0.581 s^−1^	
K_m_${\text{NO}}_{2}^{-}$	251 μM	
K_m_O_2_	24.3 μM	

## Results

**Figure 1:**
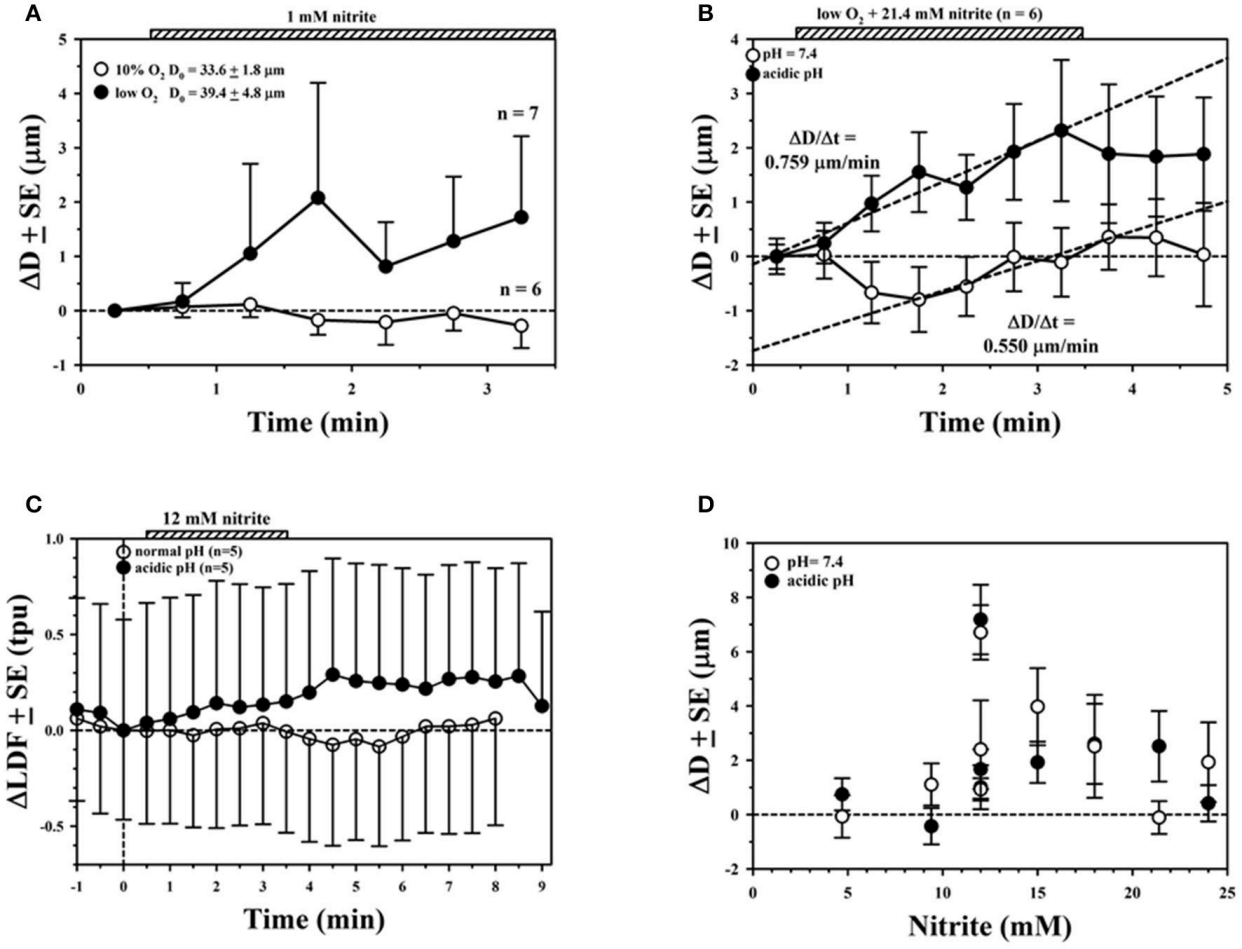
Average ± SE changes in arteriolar diameter (ΔD) measured in rat mesentery during superfusion experiments. **(A)** No effect was seen with 1 mM sodium nitrite (NaNO_2_) in 10% oxygenated solution (open circles), whereas increases in D were observed with hypoxic solution (solid circles). **(B)** Much larger and more rapid changes in D with NaNO_2_ were often observed with acidic solution (solid circles) than solution with normal pH (open circles). **(C)** Changes in laser Doppler signal (LDF) showing larger response with acidic superfusion solution. **(D)** Overall ΔD ± SE during superfusion with normal (open circles) or acidic pH (solid circles) over a wide range of NaNO_2_ concentrations.

The presence of NaNO_2_ in the superfusion medium usually elicited vasodilatory responses for the arterioles observed in this study, consistent with an increase in NO bioavailability. In a few cases, no change or minor vasoconstriction was observed. However, we were not able to accurately measure perivascular tissue NO due to electrochemical interference with nitrite at the high concentrations used in our superfusion experiments. Quantitative results using superfusion protocols are summarized in Figure 1 for several individual experiments using short exposures to NaNO_2_ (typically 3 min duration, indicated by striped bars). There were approximately 30 s transport delays from the time when the superfusion pump was switched between reservoirs at *t* = 0 to the time that changes in concentration reached the tissue. The transport delay was determined by observing the time for a bubble introduced at the inlet to emerge at the outlet of the tubing. Representative changes in arteriolar diameter (ΔD) are shown in Figure 1A with average responses for 6–7 arterioles from 1 rat experiment, demonstrating enhanced vasodilation with hypoxic conditions in the superfusate. Initial diameters ± SE are indicated. Greater vasodilation responses to NaNO_2_ were often, but not always, observed using hypoxic solutions with acidic pH compared with hypoxic solutions at normal pH = 7.4. An example from 1 rat experiment with averaged measurements from 6 arteriole pairs is shown in Figure 1B. Note that the time rate of diameter change (ΔD/Δt, dashed lines) during the period of NaNO_2_ superfusion was 38% faster in the acidic solution (0.759 μm/min) compared with the rate of increase at pH = 7.4 (0.55 μm/min) that occurred following a transient decrease in ΔD for this experiment. This transient may reflect a decrease in NO during the period of hypoxic superfusion preceding the exposure to NaNO_2_. We also recorded the laser Doppler signal (LDF), which is proportional to capillary blood flow and reported with arbitrary tissue perfusion units (tpu). For the example shown in Figure 1C, the average change (ΔLDF) during NaNO_2_ superfusion was negligible when the superfusate pH = 7.4 (open circles). There were increases in capillary blood flow during NaNO_2_ superfusion with acidic pH (Figure 1C, solid circles), although the difference compared with normal pH was not statistically significant. Overall results for the average ΔD ± SE for 44 paired arterioles from *n* = 9 rats are shown in Figure 1D for NaNO_2_ concentrations ranging between 4 and 24 mM in hypoxic superfusion solution. The average ± SE initial diameter was D_initial_ = 43.4 ± 1.6 μm for these measurements. Note that there were many experiments (4/9) where the average increase in ΔD with NaNO_2_ in acidic superfusion solution was smaller compared to superfusion solutions at normal pH. In three experiments, the average D was slightly negative (no increase in D for two experiments with pH = 7.4 and one experiment for acidic pH). Consequently, there was no statistically significant difference in ΔD determined during NaNO_2_ superfusion with normal or acidic pH (Mann–Whitney Rank Sum test).

**Figure 2:**
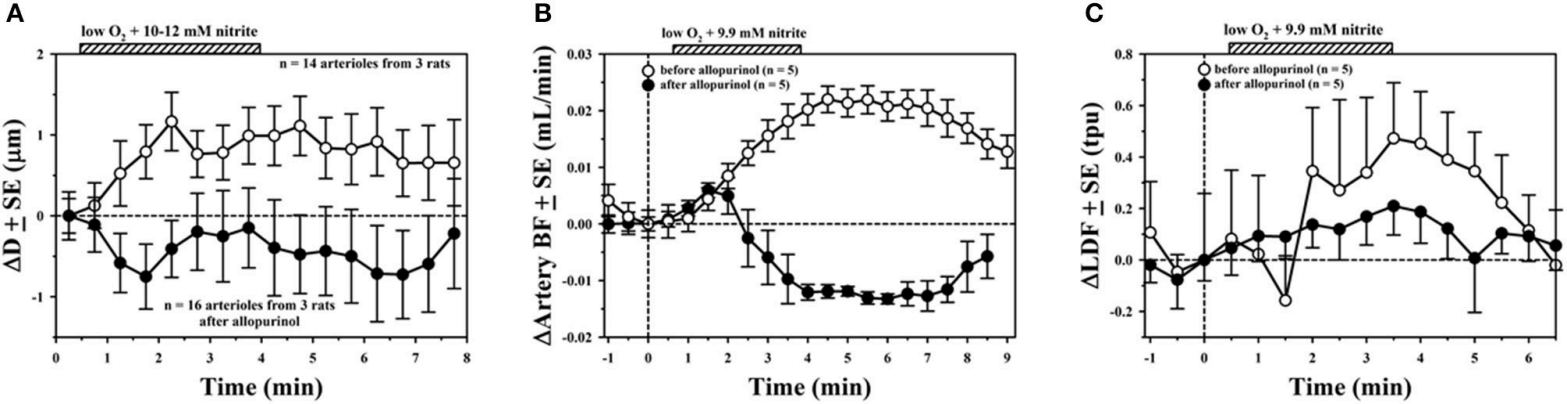
Average ± SE changes before (open circles) and after inhibiting XOR with allopurinol (solid circles) in **(A)** arteriolar diameter, **(B)** small artery blood flow, and **(C)** laser Doppler signal.

Evidence for the role of XOR was found by comparing vascular responses before and after treating animals with allopurinol (3.4–6 mg/kg IP). Results for the average ΔD with hypoxia and NaNO_2_ in the 10–12 mM range for three rat experiments is shown in Figure 2A, demonstrating a significant reduction in the vasodilatory response after allopurinol (solid circles) compared to control measurements (open circles). The small artery blood flow (BF) to the segment of mesentery under study in a representative experiment (Figure 2B) was also affected by NaNO_2_ and hypoxic superfusion, presumably due to downstream vasodilation. There was a prolonged increase in BF that persisted for several minutes after 3 min exposure to NaNO_2_, which was abolished after allopurinol treatment. Capillary perfusion as determined by LDF for this same experiment showed a similar increase with NaNO_2_ and hypoxia that was attenuated after allopurinol (Figure 2C).

**Figure 3:**
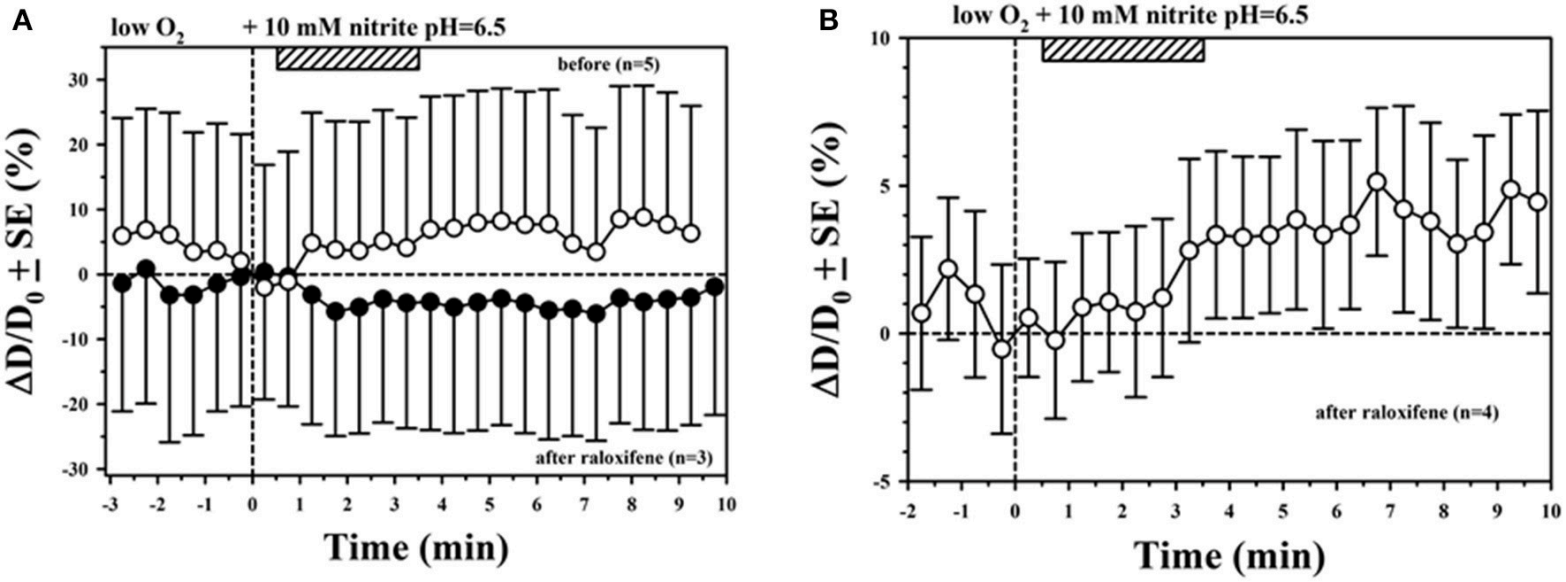
Mixed results for inhibition of AOR with raloxifene. **(A)** Inhibition of vasodilation with superfused NaNO_2_ was observed in this experiment. **(B)** Vasodilation with superfused NaNO_2_ was still observed after treating with raloxifene in this experiment.

We also investigated the role of AOR using raloxifene (2.9–10 mg/kg IP) to inhibit its activity. An example using superfusion with 10 mM NaNO_2_ in hypoxic and acidic solution is shown in Figure 3A, showing vasodilation before treatment (open circles), and complete blocking of the response after raloxifene treatment (solid circles). However, in another experiment shown in Figure 3B, treatment with raloxifene did not abolish the vasodilatory response to NaNO_2_ in hypoxic and acidic solution.

**Figure 4:**
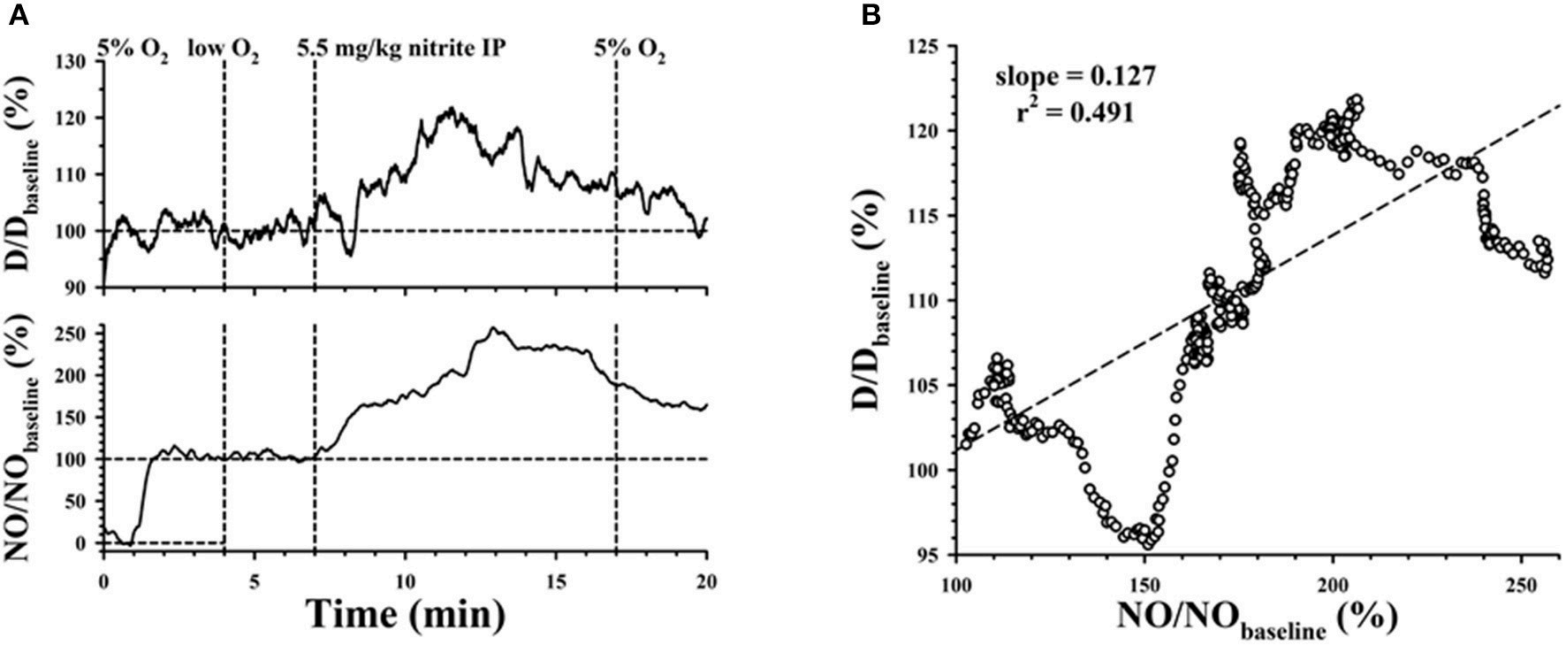
**(A)** Simultaneous measurements of arteriolar diameter and perivascular NO, normalized by baseline diameter and electrode current. Superfusion was changed from 5% O_2_ to low O_2_ at *t* = 4 min, then back to 5% O_2_ at *t* = 17 min, following an IP injection of NaNO_2_ at *t* = 7 min. **(B)** Correlation between the normalized perivascular NO and normalized diameter.

In addition to superfusion experiments, we also quantified the effect of NaNO_2_ delivered to the animal by IP injection. NO microelectrodes were used in these experiments to measure perivascular NO for the arterioles since there was no nitrite in the superfusion solution to interfere with the electrochemical measurement. A representative NO microelectrode measurement is shown in the lower panel of Figure 4A. At *t* = 0, the NO microelectrode tip was in the superfusate flowing above the preparation, where there is negligible NO concentration with resulting minimum electrochemical current. The tip was then moved close to the outer surface of the arteriole to measure the baseline perivascular NO, which was used to normalize the measurement. At *t* = 4 min, the superfusate was changed from a solution with 5% O_2_ and 5% CO_2_ to a hypoxic solution equilibrated with 0% O_2_ and 5% CO_2_. At approximately *t* = 7 min, 5.5 mg/kg of NaNO_2_ was injected IP. Relative changes in diameter, normalized to the baseline diameter, are shown in the upper panel of Figure 4A. The peak change in NO occurs about 6 min afterwards, with the peak change in D around 5 min after IP injection. Nevertheless, time courses for the relative changes in NO and D were similar. The correlation between the relative increase in D with NO for normalized data between 7 and 13 min is shown in Figure 4B, with positive slope = 0.127%/%. At *t* = 17 min, the superfusion was changed back to oxygenated solution (5% O_2_, 5% CO_2_) and the arteriolar diameter returned close to baseline, although NO remained elevated for this example. At the end of each measurement, the microelectrode tip was drawn back up into the superfusate to obtain another zero NO current measurement. Any change in the zero NO current from the beginning to the end of the measurement was used to correct for drift, assuming a linear time course.

**Figure 5:**
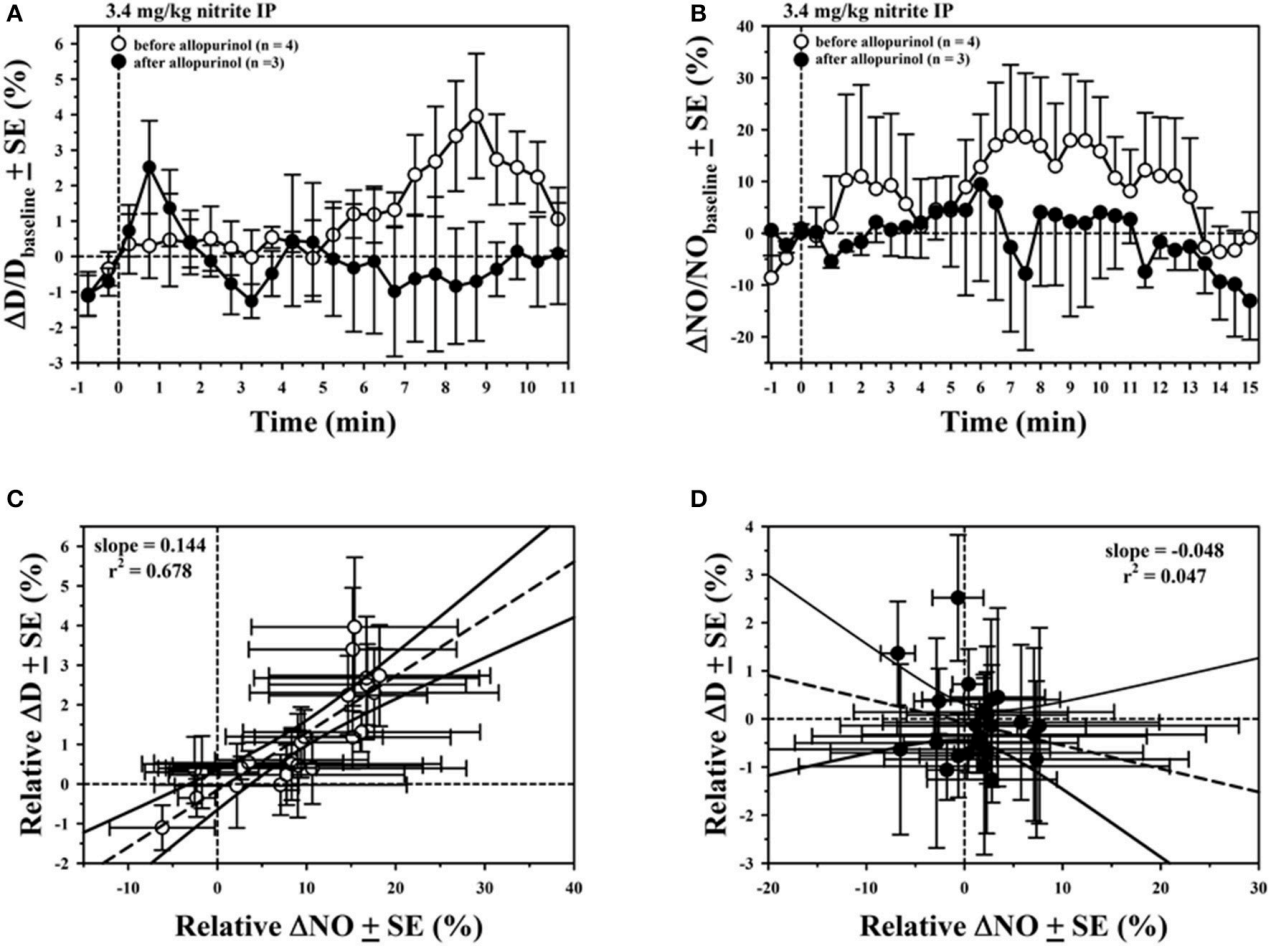
Average ± SE changes in **(A)** normalized arteriolar diameter, **(B)** normalized perivascular NO, following IP injection of NaNO_2_ at *t* = 0 before (open circles) and after inhibiting XOR with allopurinol (solid circles). Correlations between normalized perivascular NO and normalized diameter are shown **(C)** before and **(D)** after allopurinol.

Further evidence for the role of XOR was found from experiments where nitrite in the bloodstream was increased following IP delivery. Microelectrode measurements confirmed that there was an increase in perivascular NO after IP delivery of NaNO_2_, which was attenuated after allopurinol. Examples for the time course of relative average changes in diameter (Figure 5A) and perivascular NO (Figure 5B) for 7 arterioles from 1 rat experiment are shown following IP delivery of 3.4 mg/kg NaNO_2_ before and after allopurinol. The ΔD are normalized with respect to the initial diameter (D_initial_ = 37.0 ± 1.9 μm) and ΔNO with respect to the baseline NO level before each nitrite injection. Before allopurinol, the peak change in D after IP injection of NaNO_2_ occurred around 8.5 min in Figure 5A, and around 5 min in Figure 5B. There is a significant positive correlation (slope = 0.144%/%) between the normalized average ΔD and ΔNO (Figure 5C) with NaNO_2_ before inhibiting XOR. After a single dose of allopurinol (5 mg/kg IP), there is no longer any correlation (slight negative slope = −0.048%/%, Figure 5D). A total of *n* = 3 rat experiments using IP NaNO_2_ delivery were conducted with allopurinol, and in all three cases, a positive correlation between ΔD and ΔNO was observed before allopurinol (*n* = 11 arterioles) and a negative correlation between ΔD and ΔNO was observed after allopurinol (*n* = 9 arterioles).

### Model predictions

**Figure 6:**
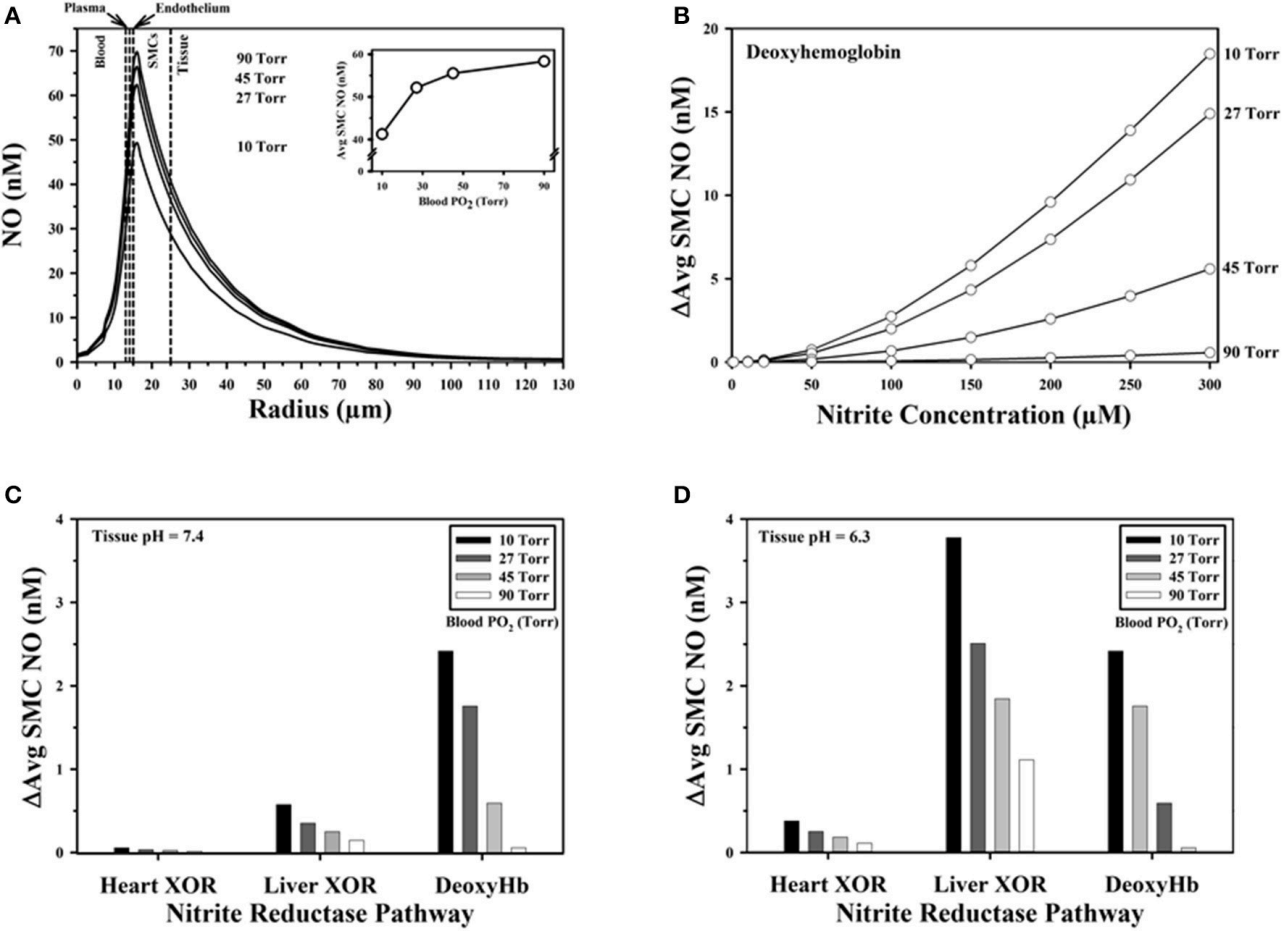
Model predictions. **(A)** Baseline NO concentration profiles across the computational domain predicted for different blood PO_2_ values without additional NO released from nitrite. Vertical dashed lines mark boundaries between the five radial model layers. (Inset) Average NO concentration in the smooth muscle cell (SMC) region with decreasing blood PO_2_. **(B)** Increase in average SMC NO above baseline predicted with formation of N_2_O_3_ from deoxyhemoglobin nitrite reductase activity and subsequent release of NO shown as a function of blood PO_2_ and nitrite concentration in blood. Effect of low (e.g., in heart) and high (e.g., in liver) tissue nitrite reductase XOR concentrations on elevation of average SMC NO above baseline for **(C)** pH = 7.4 and **(D)** pH = 6.3.

Reducing values for blood PO_2_ in the simulation from normoxic (90 Torr) to severely hypoxic levels (10 Torr), predicts a decrease in NO across the computational domain for a baseline case without any generation of NO from nitrite either in blood or tissue (Figure 6A). The average NO in the SMC layer decreases monotonically with increasing hypoxia (inset, Figure 6A), predicting a 17.1 nM decrease in NO (−29.3%) as blood PO_2_ drops from 90 to 10 torr. As reviewed by Gladwin et al. (2009), changes in conformation as hemoglobin becomes deoxygenated results in changes in the rate of nitrite reduction, with a maximum rate in the hypoxic PO_2_ range. Our previous simulations (Liu Y. et al., 2016) demonstrate that SMC NO can be significantly elevated through the deoxyhemoglobin nitrite reductase pathway, and further increased in magnitude with increasing nitrite concentration and greater hypoxia. For the case where blood PO_2_ drops from 90 to 10 torr, the decrease in SMC NO can be compensated through the deoxyhemoglobin nitrite reductase pathway by increasing the blood nitrite concentration to 284.9 μM (Figure 6B).

The effect of tissue XOR nitrite reduction on SMC NO with tissue nitrite = 100 μM was compared against the baseline case (zero nitrite reduction in Figure 6A) as a function of blood PO_2_ for low (0.03 μM) and high (0.3 μM) concentrations of XOR (Figures 6C,D). The contribution to SMC NO from the deoxyhemoglobin nitrite reductase pathway is also shown. NO elevation by XOR has the greatest effect with the highest XOR concentrations at the acidic pH and lowest blood PO_2_. For this concentration of nitrite (100 μM), the additional NO is predicted to be +2.4 nM from deoxyhemoglobin nitrite reductase and +3.8 nM from XOR, for a total compensation of +6.2 nM, representing a recovery of 36% from the drop in NO due to the decrease in blood PO_2_. The total compensation in NO at pH = 7.4 with the 0.3 μM XOR concentration would be only +3 nM (17.5% recovery), and only +2.47 nM (14.4% recovery) with the lower 0.03 μM XOR concentration. Simulations were also run for the AOR nitrite reductase pathway. AOR has slightly lower reaction rate constants compared with XOR (Maia et al., 2015), and AOR concentrations are generally lower than XOR in the heart and liver (Li et al., 2008). Simulations with AOR predicted ~10–20% less elevation in SMC NO for pH = 7.4 and 6.3 (not shown).

**Figure 7:**
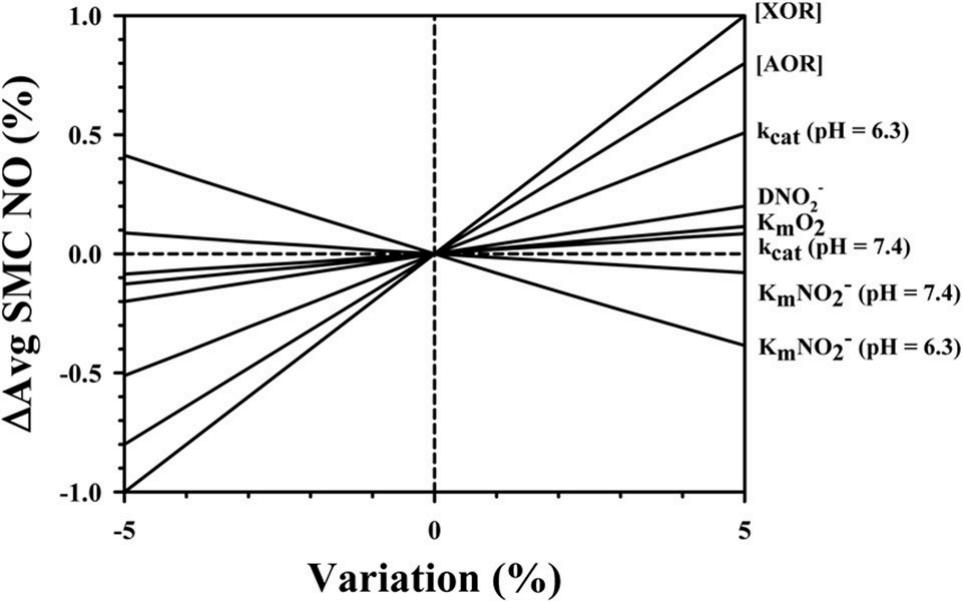
Sensitivity analysis showing the effect of ±5% variations in the model parameters listed in Table 1 (identified at right of figure) on relative differences in average NO in the SMC region.

A sensitivity analysis (Figure 7) was conducted for the model parameters listed in Table 1 to examine the effect of small variations (±5%) in the parameter values on the predicted average SMC NO. Other parameters were held constant for simulations with tissue nitrite = 100 μM, blood PO_2_ = 10 Torr, and baseline flow in liver tissue (Figure 7). Variations in the concentration of XOR or AOR had the largest effect on the predicted NO, with an intermediate sensitivity to the nitrite diffusion coefficient. The sensitivity was relatively low (<0.1% at +5% variation) for K_m_O_2_, and the reaction parameters k_cat_ at pH = 7.4. There was a larger effect for variations in the reaction parameter k_cat_ at pH = 6.3 (+0.508% at +5% variation). A 5% increase in the values for K_m_${\text{NO}}_{2}^{-}$ at pH = 7.4 and K_m_${\text{NO}}_{2}^{-}$ at pH = 6.3 results in small decreases in the SMC NO by −0.079 and −0.385%, respectively.

## Discussion

Our *in vivo* results from the rat mesentery microcirculation provide further evidence that nitrite reductases in tissue play a role in increasing NO bioavailability during nitrite-mediated hypoxic vasodilation. We confirmed this by using allopurinol to inhibit XOR (3.4–6 mg/kg IP). Due to time constraints for the *in vivo* experimental procedure, we did not investigate whether a second, higher dose of allopurinol would further inhibit the vasodilatory response. Golwala et al. (2009) used a higher dose of allopurinol (25 mg/kg IV) for their *in vivo* rat studies, and demonstrated that a second 25 mg/kg dose had little further inhibitory effect. They also inhibited AOR using cyanamide (25 mg/kg IV), and alternated the order of inhibitor delivery to discriminate between the contribution of each nitrite reductase on nitrite responses. Nitrite doses of 0.01, 0.03, or 0.1 mM/kg were delivered IV and the changes in systemic blood pressure were measured. Both inhibitors attenuated the decrease in systemic blood pressure with IV delivery of NaNO_2_. The authors concluded that both XOR and AOR pathways act in parallel in the vasculature.

In our study, we found that it was necessary to use high NaNO_2_ concentrations (>1 mM in the superfusion solution) to elicit vasodilation (Figure 1D). A possible explanation for this finding is that the hypoxic superfusion solution did not significantly lower the blood PO_2_ of the arterioles under study, even though we did see a difference (greater vasodilation) with NaNO_2_ in hypoxic solution compared with NaNO_2_ in oxygenated solution (Figure 1A). The high NaNO_2_ concentration used in our superfusion experiments is much greater than used for intra-arterial infusions in humans, which demonstrate greater vasodilation under hypoxia than normoxia (Maher et al., 2008). The necessity to use high concentrations of nitrite to generate NO under anoxic conditions *in vitro* was pointed out in the review by Kelley (2015). Consequently, we were not able to directly measure perivascular NO due to electrochemical interference with high nitrite concentrations during superfusion, but we were able to confirm that there was an increase in perivascular NO from the measurements using IP delivery of nitrite. A direct correlation between the increase in NO and vascular diameter was found from these IP injection experiments, which was essentially abolished after inhibiting XOR. The review by Kelley (2015) identifies key factors which allow significant recovery of NO through XOR, including acidic pH and low O_2_, as well as other biochemical factors. For example, a recent *in vitro* study using aortic ring preparations and isolated mesenteric arterial bed perfusion found that the nitrate anion (${\text{NO}}_{3}^{-}$) attenuates XOR-mediated NO generation from nitrite (Damacena-Angelis et al., 2017). To validate the inhibitory effects of nitrate, this study also included experiments with purified XOR using a different inhibitor (febuxostat), which is more potent than allopurinol (Okamoto et al., 2003).

Bryan et al. (2005) studied the time course for uptake and metabolism of nitrite in different organ systems of Wistar rats following IP injection of NaNO_2_ (range 0.1–10 mg/kg), reporting that tissue nitrite levels were essentially in equilibrium by ~5 min, when presumably the uptake rate from the abdominal cavity matches the decay rate in blood. Our measurements for the maximum ΔD and ΔNO for the examples shown (Figures 4A, 5) ranged between 5 and 8.5 min. Bryan et al. (2005) reported that nitrate levels as well as the total nitroso/nitrosyl products (RSNO + RNNO + NO-heme) also increased following IP injection of nitrite. We cannot rule out the possibility that some inhibition of the vascular responses by nitrate might occur after repeated IP injections of nitrite in our study as suggested from the results reported by Damacena-Angelis et al. (2017). Bryan et al. (2005) also conducted *in vitro* experiments, and presented evidence that NO formation from nitrite is not required for nitrosation (RSNO) or nitrosylation (RNNO) of thiols, and conclude that these reactions can occur at nitrite concentrations far below that required for vasodilation. A randomized, placebo controlled dose-response study of NaNO_2_ infusion in humans by Rosenbaek et al. (2017) investigating effects on kidney function and blood pressure found a dose-dependent decrease in urine output with reduced blood pressure. Since they observed no increase in GMP, they concluded that their results supported a direct effect of nitrite or nitrate on the renal tubules and vascular bed with little or no systemic conversion of nitrite to NO.

Many previous studies that have examined the conversion of nitrite to bioactive NO have focused on the role of RBCs through the reductase activity of deoxyhemoglobin (e.g., see Kim-Shapiro and Gladwin, 2014). However, we cannot discriminate whether our experimental results can be specifically attributed to nitrite reductases in blood or tissue (or both). There is strong evidence for greater involvement of tissue nitrite reductases (Feelisch et al., 2008; Li et al., 2008; Arif et al., 2015; Piknova et al., 2015). *In vitro* studies with isolated rabbit aortic rings demonstrate that 10 μM nitrite in the absence of hemoglobin can increase maximal dilation under hypoxic conditions, which can occur with or without the endothelium (Pinder et al., 2009). The authors concluded that AOR, but not XOR, was primarily responsible for nitrite-mediated hypoxic vasorelaxation measured in their study, with some contribution from the cyclooxygenase (COX) pathway. Another *in vitro* study with isolated rat thoracic aorta rings demonstrated attenuation of hypoxic vasorelaxation with nitrite (concentration range from 1 nM to 100 μM) after inhibiting AOR with cyanamide (Arif et al., 2015). We did not get consistent results inhibiting vascular responses to nitrite with raloxifene. We found nitrite-mediated vasodilation was unaffected after raloxifene in one study (Figure 3B). Since there is evidence that high concentrations of raloxifene can also inhibit XOR (Weidert et al., 2014), we cannot rule out the possibility that our results showing inhibition of vascular responses with raloxifene (Figure 3A) might be due to inhibition of XOR instead of AOR. There might also be vasoactive effects of raloxifene that are independent of NO. We estimated that the raloxifene dose chosen for our studies was not high enough to inhibit XOR. However, it is difficult to discriminate between NO contributions between XOR and AOR pathways since blocking either pathway depends on inhibitor specificity and dose (Weidert et al., 2014).

It should be recognized that there are other possible mechanisms for the RBC to contribute to hypoxic vasodilation besides the deoxyhemoglobin nitrite reductase-mediated release of NO from nitrite. It has been proposed that the RBC contains a form of eNOS (Kleinbongard et al., 2006), which can produce NO, although it would be subject to immediate scavenging due to the high Hb concentration in the RBC. It has been proposed that formation of Hb(III)NO as an intermediate can account for the majority of NO produced from RBCs (Nagababu et al., 2003). Salgado et al. (2015) propose that this intermediate preferentially locates to the RBC membrane with a greater affinity than Hb. They suggest that a significant amount of NO might be transferred to the vasculature from this pool, avoiding quenching by Hb. Alternately, there is evidence that the RBC releases ATP during hypoxia, which can in turn stimulate NO production by eNOS (Sprague et al., 2007; Cao et al., 2009). We have modeled this effect to predict how ATP can increase shear-stress mediated NO production (Kirby et al., 2016), based on *in vitro* NO measurements with cultured ECs (Andrews et al., 2014). Another proposed mechanism is the formation of S-nitrosohemoglobin (SNO-Hb), as reviewed by Singel and Stamler (2005). However, there is contradictory evidence for this mechanism (Isbell et al., 2008). Furthermore, a mathematical model by Chen K. et al. (2007) did not predict a significant NO contribution from this pathway.

There are also other mechanisms in tissue beside nitrite reductases that can contribute NO during hypoxia, as reviewed by Buerk (2007) and Kim-Shapiro and Gladwin (2014). There is evidence that cytochrome c in the mitochondria can be a source of NO by reducing nitrite (Kozlov et al., 1999). It has also been proposed that there is a mitochondrial form of NOS (mtNOS) that can produce NO, although the existence of mtNOS is questioned (Lacza et al., 2006). NO or related reactive species can modify tissue proteins, forming *S*-nitrosothiols, *S*-nitrosoalbumin, and other *S*-nitrosoprotein species under normal physiological conditions which might serve as a storage pool in tissue for NO or other vasoactive species (Jourd'heuil et al., 2000; Liu T. et al., 2016). On the other hand, these reactions may modify vascular tone signaling pathways independently from any NO recovered from nitrite. While normally myoglobin is a strong scavenger of NO, it is recognized that myoglobin can also cause nitrite bioactivation by reducing nitrite (Shiva et al., 2007; Totzeck et al., 2012, 2014; Piknova et al., 2015). We have developed a mathematical model for a cardiac arteriole and surrounding myocardium (Liu et al., 2017), showing how myoglobin functions as a nitrite reductase to elevate SMC NO during hypoxia in an O_2_- and pH-dependent manner.

### Limitations of mathematical model

Our simulations assume that published reaction parameters (Maia and Moura, 2011; Maia et al., 2015) were constant with abundant substrate, and were only affected by changing O_2_ or pH levels. The model assumes that all N_2_O_3_ homolyzes to NO, ignoring other nitrosation reactions that are known to occur (Basu et al., 2007; Kim-Shapiro and Gladwin, 2014), thus we may be overestimating the increase in SMC NO. It is also possible, as suggested by Koppenol (2012) and Tu et al. (2009), that it is not energetically possible for the reactions to generate N_2_O_3_ to occur. As with any computer simulation, the accuracy of model parameters determines whether the predictions are physiologically relevant. The measurement of reaction rates is hindered by experimental difficulties and complex interactions among XOR, AOR, nitrite, nitrate, and NO (Jourd'heuil et al., 2000; Maia and Moura, 2011; Cantu-Medellin and Kelley, 2013; Damacena-Angelis et al., 2017). The concentration of nitrite reductase enzymes in tissue is not well-characterized and may not be uniformly distributed. We are not aware of any estimates for the enzyme concentrations and reaction parameters in mesentery. Future simulations would be necessary if more precise information about reaction rates, localized spatial distributions, and concentrations become available. The predicted changes in SMC NO with physiologically relevant nitrite concentrations in the 0.2–2 μM range are quite small (<1 nM) even for hypoxic conditions. Even with much higher nitrite levels (up to 300 μM), the increase in SMC NO predicted for the deoxyhemoglobin nitrite reductase pathway is <20 nM during severe hypoxia with blood PO_2_ = 10 torr (Figure 6B).

In conclusion, our *in vivo* experimental results are consistent with other studies that suggest that the reduction of nitrite through the activity of tissue nitrite reductases can contribute NO to vascular smooth muscle to enhance hypoxic vasodilation. However, it is difficult to assess whether hypoxic vasodilation can be specifically attributed to nitrite reductase activity in blood or tissue, or both. It is also very difficult to experimentally create the severe *in vivo* physiological conditions for maximal effects. Our theoretical model demonstrates that is possible to compensate for the loss of NO production by eNOS during hypoxia by all three pathways: deoxyhemoglobin nitrite reductase in RBCs, and the tissue nitrite reductases XOR and AOR. This modeling approach can be further developed to explore other biochemical pathways that can contribute NO or other nitrogen species that enhance hypoxic vasodilation.

## Author contributions

DB was responsible for the experimental design, conducting animal research, data analysis, model development, writing and editing the paper. YL was responsible for computer simulations and contributed written content. KZ assisted with animal surgery and editing the paper. KB and DJ were responsible for model development and editing the paper.

### Conflict of interest statement

The authors declare that the research was conducted in the absence of any commercial or financial relationships that could be construed as a potential conflict of interest.
